# An Optical Biosensor from Green Fluorescent *Escherichia coli* for the Evaluation of Single and Combined Heavy Metal Toxicities

**DOI:** 10.3390/s150612668

**Published:** 2015-05-28

**Authors:** Dedi Futra, Lee Yook Heng, Asmat Ahmad, Salmijah Surif, Tan Ling Ling

**Affiliations:** 1 School of Chemical Science and Food Technolgoy, Faculty of Science, Universiti Kebangsaan Malaysia, 43600 UKM Bangi, Selangor D.E., Malaysia; E-Mails: futra.dedi@yahoo.com (D.F.); asmat@ukm.edu.my (A.A.); salmijahsurif@gmail.com (S.S.);; 2 Southeast Asia Disaster Prevention Research Initiative (SEADPRI-UKM), LESTARI, Universiti Kebangsaan Malaysia, 43600 UKM Bangi, Selangor D.E., Malaysia; E-Mail: babybabeoo@gmail.com

**Keywords:** whole cell biosensor, hazardous heavy metal, optical fiber biosensor, GFP *E. coli*

## Abstract

A fluorescence-based fiber optic toxicity biosensor based on genetically modified *Escherichia coli* (*E. coli*) with green fluorescent protein (GFP) was developed for the evaluation of the toxicity of several hazardous heavy metal ions. The toxic metals include Cu(II), Cd(II), Pb(II), Zn(II), Cr(VI), Co(II), Ni(II), Ag(I) and Fe(III). The optimum fluorescence excitation and emission wavelengths of the optical biosensor were 400 ± 2 nm and 485 ± 2 nm, respectively. Based on the toxicity observed under optimal conditions, the detection limits of Cu(II), Cd(II), Pb(II), Zn(II), Cr(VI), Co(II), Ni(II), Ag(I) and Fe(III) that can be detected using the toxicity biosensor were at 0.04, 0.32, 0.46, 2.80, 100, 250, 400, 720 and 2600 μg/L, respectively. The repeatability and reproducibility of the proposed biosensor were 3.5%–4.8% RSD (relative standard deviation) and 3.6%–5.1% RSD (n = 8), respectively. The biosensor response was stable for at least five weeks, and demonstrated higher sensitivity towards metal toxicity evaluation when compared to a conventional Microtox assay.

## 1. Introduction

Toxic pollution occurs when hazardous chemicals are discharged and spread throughout the world, due to the rapid pace of industrial development. Toxicants that are widely found in waterways are heavy metals, herbicides, and pesticides, due to the extensive application of these hazardous chemicals in agricultural industries and chemical processing plants. There are more than 100 toxicants (metals) in the environment having a detrimental effect on human and biological systems, and 23 of these are heavy elements. Heavy metal contaminants are comprised of metals and metalloids with atomic densities that are higher than water (>4 g/cm^3^) [1,2]. At low metal concentrations, they are not detrimental to human health and biological systems, as a matter of fact; they are essential towards a healthy life e.g., iron, copper, manganese, and zinc. These elements are functionalized as catalysts of biochemistry reactions in cells, enzyme stability, regulation of gene expression, and regulatory of osmosis pressure for membranes [3]. High toxicant concentration can be toxic to human and biological systems, as metals are non-biodegradable. For instance, lead (Pb) is suspected of causing neurological damage, leading to reduction in intelligence and loss of memory. High levels of lead are a big problem in urban areas. In Brazil, there are approximately 80% of children with blood lead concentrations exceeding 10 µg/dL [4]. Therefore, it is necessary to develop sensitive, effective, and inexpensive methods for monitoring the concentration of heavy metals in the environment.

Inductively coupled plasma-mass spectrometry (ICP-MS) [5] and atomic absorption spectrometry (AAS) have been widely used for heavy metal detection in environmental samples [5,6]. However, these methods are not able to identify toxicity levels with regard to metals, they can only be used to determine the concentration of a particular metal [7]. Biotests based on organisms for heavy metal toxicity assay, on the other hand, involve organisms that are normally not physically protected by biofilms, and are directly in contact with toxicants such as *V. fischeri* [8,9], *J. Lividium* [9], *P. fluorescence* [10], and *D. magna* [11]. These biotests require extended analysis time (hours), but less sensitivity, and give high EC_50_ values at ppm level. Recently, recombinant bacteria have been extensively used for bioassay to determine the levels of Pb(II) [7,12,13], Cd(II) [7,13,14], Cu(II) [13,15], and Zn(II) toxicities [13,15]. *E. coli* (Alux gene) has been utilized to evaluate Zn(II) [16,17], Cd(II) [16], Hg(I) [16,17,18], and As(II) toxicities [17]. The bioassays based on recombinant bacterial cells were found to be effective for the assessment of heavy metal toxicity in water [12], sediment, and soil samples [7].

In this paper, we describe the fabrication of toxicity biosensor using recombinant green fluorescent protein (GFP) *E. coli* immobilized on the cellulose nitrate membrane, covered with a layer of Ca-alginate membrane for the detection of Cu(II), Cd(II), Pb(II), Zn(II), Cr(VI), Co(II), Ni(II), Ag(I), and Fe(III) toxicities. This work is focused only on evaluation of the toxicity of single and mixed heavy metals and may be applicable to the determination of the toxicity of real and complex aqueous samples. The bacteria to our knowledge has not been explored for heavy metal toxicity evaluation. There is a difference between this work and the *A. fischeri* based toxicity biosensor reported earlier [19]. The immobilization method is different from the previous reported biosensor, *i.e.*, alginate micropshere encapsulation *versus* alginate coated cellulose membrane used in this work. The GFP used in this work has been reported before. Bomati *et al.*, [20] studied the comparison of spectral data and structure between bright and dim GFP in amphioxus. Segami *et al.*, [21] reported the dynamics of vacuoles and H^+^-pyrophosphatase visualized by monomeric GFP in Arabidopsis. Several applications of GFP including enzyme immobilization in food processing and biotechnological industries [22] and screening of multiple membrane proteins in *E. coli* [23]. In short, GFP-modified cells were utilized as a biomarker because they can be employed without the need of exogenous substrate. Several advantages are high stability, suitable pH range at pH 6–10, and temperature of up to 60 °C for general operations.

## 2. Materials and Methods

### 2.1. Materials

All chemicals used were of analytical grade. Stock solutions of Cadmium(II), Lead(II), Zinc(II), and Copper(II)) were prepared from their respective chloride salts (Sigma, St. Louis, MI, USA). Luria Bertani medium (trypton, yeast extract, sodium chloride), cobalt(II) nitrate (Co(NO_3_)_2_, iron(III) nitrate anhydrate (Fe(NO_3_)_3_·9H_2_O), silver nitrate (AgNO_3_), and nickel(II) nitrate (Ni(NO_3_)_2_) were purchased from BDH (Radnor, PA, USA). Hydrogen chloride (HCl), sodium hydroxide (NaOH), 4-(2-hydroxyethyl)-1-piperazineethanesulfonic acid (Hepes) buffer, alginate acid, calcium chloride (CaCl_2_), and potassium dichromate (K_2_Cr_4_O_7_) were obtained from Sigma. All glassware used was cleaned by immersion in nitric acid (15%) for 24 h to remove trace elements, and sterilized by autoclaving at 121 °C for 20 min.

### 2.2. GFP-Modified E. coli Bacteria

The gene of the recombinant GFP *E. coli* was constructed by introducing a His-tagged version of wild-type (Q80R) GFP in the plasmid pRSETB. The mutations C48S/T65S/S147C/Q204C (which contains S65T) and C48S/S147C/Q204C were inserted into the plasmid pEGFP-N1 for expression in the bacteria cells. The constructed GFP were sub-cloned into pRSETB using BamHI and EcoRI restriction sites. Then, the subcloned pRSETB was expressed in the DH5α™ strain of *Escherichia coli* (Invitrogen, Tokyo, Japan) [24,25]. About 100 µg/mL of ampicillin was then added into the nutrient agar for plasmid maintenance, and subsequently mixed with the Luria-Bertani liquid to culture the *E. coli* cells.

### 2.3. GFP E. coli Bacterial Cell Culture

Single colony of GFP *E. coli* bacteria was grown in Luria-Bertani mediun (L-B; 10 g/L tryptone, 5 g/L yeast extract, 10 g/L NaCl), supplemented with 100 μg/mL ampicillin for 18 h under shaking in a rotary thermo-shaker at 250 rpm and 37 °C to obtain an optical density (OD) of 1.3 Abs at 600 nm on a UV-Vis spectrophotometer (Perkin Elmer, Billerica, MA, USA). After that, the GFP *E. coli* cells were harvested and further grown in 50 mL L-B medium under similar conditions.

### 2.4. Development of Microbial Biosensor

About 5.0 × 10^9^ CFU/mL or 29.2 mg/100 mL of GFP *E. coli* bacterial suspension with an optical density of 0.8–0.82 Abs at 600 nm in 10 mL of 5 mM Hepes buffer (pH 7.0) was directly immobilized on a cellulose nitrate membrane (0.2 µm pore size) by filtration with the aid of a milipore vacuum pump, and stored at 4 °C for 10 min. The GFP *E. coli* immobilized cellulose nitrate membrane was then smeared with a layer of 4% alginate solution and immersed into 0.15 M CaCl_2_ solution for 50 s. A layer of Ca-alginate gel formed spontaneously thereafter, and was left overnight at 4 °C. Then, the membrane was punched into circles of 5 mm diameter using a paper puncher to develop a miniature microbial biosensor. The fluorescence response of the immobilized GFP *E. coli* bacterial cells was measured with a fibre-optic fluorescence spectrophotometer (Perkin Elmer) by pointing the probe’s distal end directly above the microbial membrane.

### 2.5. Selectivity Study

All heavy metal solutions (0.1–200 μg/L) were prepared in deionized water, and blank deionized water was used as a control sample. The bacterial cell concentration was fixed at 0.8 Abs OD (600 nm) with 2 min incubation duration at room temperature. The fluorescence measurement was carried out in triplicate for each metal ion.

### 2.6. Optimization of Whole Cell Biosensor Response

The *E coli* GFP cell loading was optimized between 0.2 Abs and 1.1 Abs (OD_600_ nm), whilst the alginate concentration used was varied from 1.0% to 6.0%. The optimum pH of the whole cell biosensor was then determined by incubating the immobilized *E coli* GFP in 0.01–500 µg/L toxicant solution (e.g., Cu(II), Cd(II) and Pb(II)) from pH 5.5–9.0. The toxicant solution pH was adjusted by using 2 M NaOH and HCl. The micorbial biosensor response was collected at room temperature and 2 min after the biochemical reaction began.

### 2.7. Reproducibility and Long Term Stability of the Biosensor

The reproducibility studies were carried out in eight replicates for 20 µg/L (Pb(II), 10 µg/L Cu(II), and Cd(II) using 0.80–0.82 Abs OD_600_ nm (5 × 10^9^ CFU/mL) bacteria cell concentration at 2 min response time and room temperature. In the meantime, taking the biosensor fluorescence signal once a week over a period of ten weeks helped us assess the biosensor’s lifetime. The bacterial cell membranes were kept at 4 °C in the fridge when they were not in use.

### 2.8. Evaluation of Single and Combined Metal Toxicities via Biosensor and Microtox Assay

The biosensor inhibition response was investigated by using a single toxicant [Cu(II), Cd(II), Pb(II), Zn(II), Cr(VI), Co(II), Ni(II), Ag(I) and Fe(III)] and toxic cocktail [Cu(II), Pb(II) and Zn(II)]. The immobilized bacterial cell concentration was held constant at 5 × 10^9^ CFU/mL. The fluorescence inhibition signal from the immobilized GFP *E coli* was monitored at fluorescence excitation and emission wavelengths of 400 ± 2 nm and 485 ± 2 nm, respectively. The biosensor fluorescence response was recorded before and after exposure to toxicants. The toxicity levels of single toxicants and toxicant mixture were calculated using Equations (1) and (2), respectively.

(1) $$\%RFU=\frac{Fluorescence\;intensity\;with\;analyte}{Fluorescence\;intensity\;without\;analyte}\;\times 100\%$$

(2) $$\text{Σ}TU=\frac{A.\;EC_{50\%}mixture}{A.\;EC_{50\%}single}+\frac{B.\;EC_{50\%}mixture}{B.\;EC_{50\%}single}$$

where A and B are different metal concentrations coexisting in the toxicity mixture. EC_50_ is the effective concentration of a metal at 50% of its relative fluorescence unit (RFU). The EC_50_ parameters were evaluated by using probit regression model of Finney *et al.* [26]. This was estimated from linear regression parameters (probit equation, y = ax + b) at 95% confidence level with y and x as the observed probit and dose level of each test respectively. The values of a and b are the slope of the regression line and its intercept. The total toxicity unit of (ΣTU) = 1 indicates zero interaction additive effect between toxicants in a mixture, whilst ΣTU >1 indicates an antagonistic effect, and its additive index (AI) value can be calculated with AI = [(−1) × ΣTU + 1]. ΣTU<1 suggests a synergistic effect in the toxicity mixture, and its AI value is calculated with AI = [(1/ΣTU) − 1] [27]. Metal toxicity was also evaluated using Microtox acute toxicity tests and SDI Quick-Microtox Assay (Newark, DE, USA) for comparison.

## 3. Results and Discussion

### 3.1. Characteritics of the Whole Cell Biosensor Response

**Figure 1:**
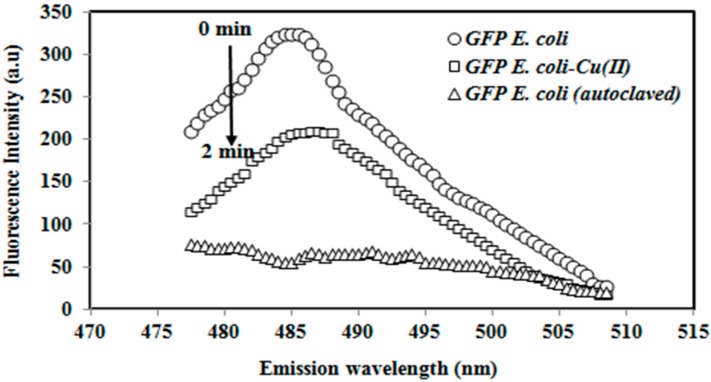
The emission spectra of the immobilized autoclaved green fluorescent protein (GFP) *E. coli* and the whole cell biosensor before and after exposure to the Cu(II) (1.0 µg/L) metal ion.

The fluorescence spectra of the autoclaved GFP *E coli* and immobilized GFP *E. coli* before and after exposure to toxicants are presented in Figure 1. In the absence of a toxicant, the immobilized GFP cell resulted in a significant fluorescence response at 485 ± 2 nm. The whole cell biosensor response declined at 485 ± 2 nm after incubation with 1.0 µg/L Cu(II) toxicant for 2 min. The reaction of Cu(II) ion with the thiol functional group of GFP inhibited the metabolism of the cell. The autoclaved GFP *E. coli* cell did not give a measurable fluorescence response due to denaturation of the GFP *E. coli* cells treated with the autoclaving process.

### 3.2. Selectivity Study

**Figure 2:**
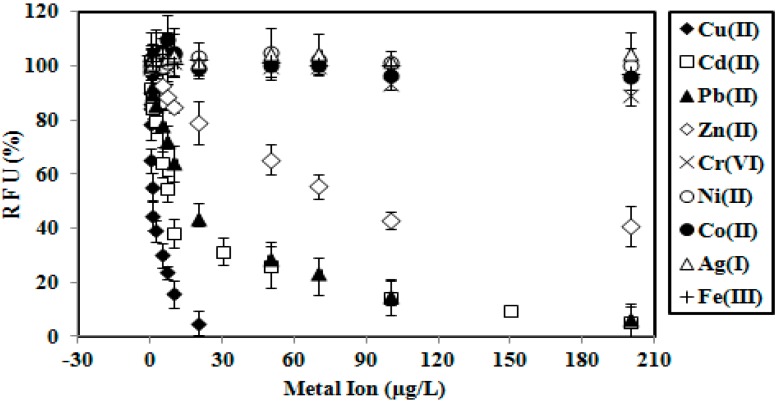
Selectivity performance of the immobilized GFP *E. coli* by Cu(II), Cd(II), Pb(II), Zn(II), Cr(VI), Co(II), Ni(II), Ag(I) and Fe(III).

For selectivity study, the biosensor was exposed to various metal ions from 0.1–200 μg/L and incubated for 2 min. The selectivity performance of the immobilized GFP *E. coli* is given in Figure 2. High responses were observed for Cu(II), Cd(II) and Pb(II) ions. The whole cell biosensor responses for Cr(VI), Ni(II), Co(II), Ag(I) and Fe(III) ions were found with low selectivities. Although the developed microbial biosensor has high selectivity towards Cu(II), Cd(II) and Pb(II), the proposed GFP cell biosensor did not specifically determine the metal ions of Cu(II), Cd(II) and Pb(II). The biosensor response was stable at maximum fluorescence intensity (100% RFU) in the absence of any toxicants. When the metal concentration was high enough, some of the toxicity biosensor responses were below 40% and decreased towards 0% (Figure 2). When the concentrations increased to much higher values, the relative fluorescence signal progressively reduced towards zero (~0% RFU), e.g., in the case of heavy metal concentration it increased from 30–200 µg/L for Cd(II) and Pb(II) and 5–20 µg/L for Cu(II) (Figure 2). This indicated that the GFP *E. coli* cells were dead and the response was likely to be related to the viability of the bacteria cells and not the GFP itself. The GFP chromophore, which plays the role of a light source forfluorescence in recombinant *E. coli* was left intact [7], whereby no interaction occurred with the thiol (S-H) functional group in the amino acid of cycteine (Cys^147^-Ser, Ser-Cys^148^, Glu-Cys^204^) [13,24,25]. In such circumstances, the thiol functional group of the chromophoric cysteine interacted with the metal ions [13]. Moreover, the lower fluorescence intensity can also be attributed to the screening effect by metal ions at the chromophore functional group [7].

The variation in biosensor response towards different metal toxicants is influenced by the mechanism of the reaction between bacterial cells and toxicants. One or more factors can be responsible for a fluorescence response of the immobilized cell to a metal ion or metal ion mixture e.g., (1) the toxicant may be shielded by membrane cell; (2) no reaction takes place between the toxicant and the target cell; (3) the toxicant reacted with functional groups other than the target functional groups inside the bacterial cell; (4) toxicants cannot enter cells as reaction occurs between the toxicant and extracellular functional groups; or (5) the toxicant is modified and does not bring the toxicity effect to the targeted cell [28]. The resistance mechanisms are organized in operon, and are usually found on plasmids carried by the resistant bacteria. The regulatory genes and promoters from the resistance operons can be used to construct promoter-reporter gene fusion for the construction of metal ion biosensors [29].

### 3.3. Optimization of Experimental Conditions

**Figure 3:**
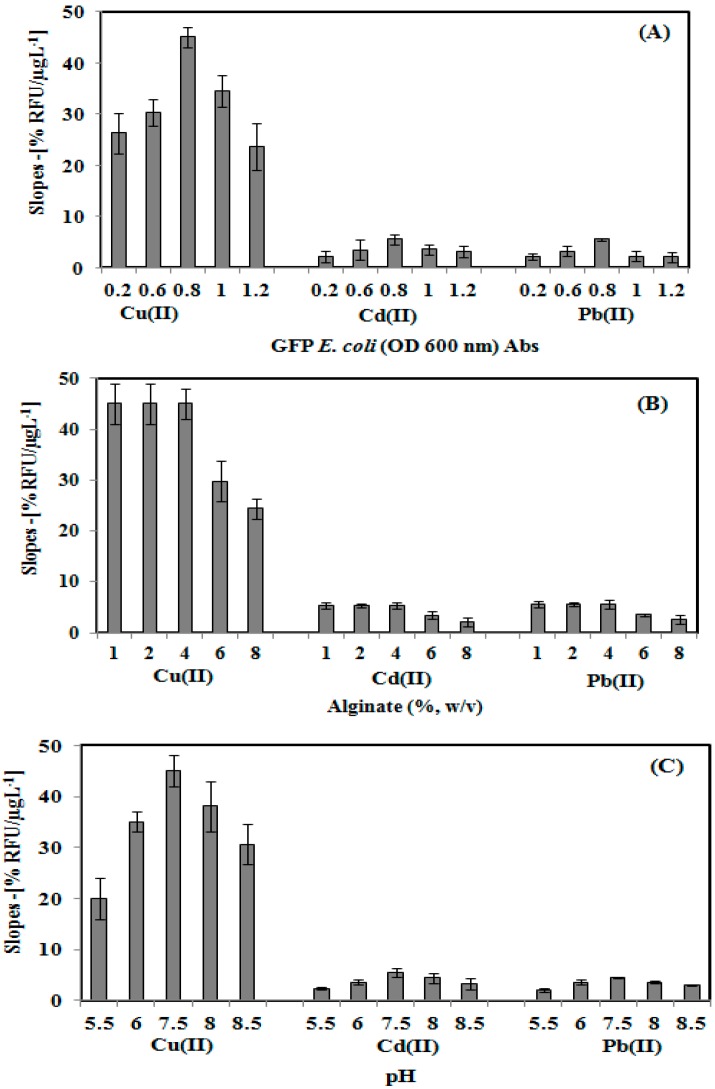
Effect of GFP *E. coli* cell loading (**A**), alginate concentration (**B**), and toxicants solution pH (**C**), on the microbial toxicity biosensor response.

The effects of GFP *E. coli* cell loading, alginate concentration, and toxicant solution pH towards the toxicity biosensor response is illustrated in Figure 3. The toxicity biosensor response increases as the *E coli* of GFP cell loading increase from 0.2–0.8 Abs due to the increasing reaction rate of the immobilized GFP *E. coli*. On further increasing the GFP *E coli* cell concentration from 0.8–1.1 Abs (Figure 3A), the toxicity biosensor response decreasesas a result of the limited diffusional transport of oxygen within the high cell density layer [19,30], leading to the fluorescence quenching of neighbouring *E. coli* cells. Therefore, the optimum GFP cells at 0.8 Abs (OD_600_ nm) was used for further evaluation of the toxicity biosensor.

Similar toxicity biosensor response trending was also obtained for the alginate concentration effect study (Figure 3B). The sensitivity of the biosensor was highly stabilized, as the alginate concentration increased from 1.0% to 4.0% (w/v). Higher alginate loading at 6.0% and 8.0% (w/v) of the GFP cell modified membrane causes the biosensor sensitivity to decrease due to the less porous structure of the gelatin system, which becomes a barrier for oxygen diffusion in the immobilized GFP cells layer. As limited oxygen interrupted the metabolism activity of the immobilized GFP cell, lower fluorescence emission was therefore obtained [31].

The pH effect towards the sensitivity of the microbial biosensor was investigated by using Cu(II), Cd(II) and Pb(II) toxicant solutions at different pHs (Figure 3C). The whole cell biosensor sensitivity increased as the pH environment increased from pH 5.5–7.5, due to the reaction between the carboxyl functional group of the alginate and metal ion to form metal hydroxide complexes under acidification conditions (<pH 6.5) [32,33]. An alkaline condition with a pH above pH 7.5 resulted in the sensitivity of the microbioal biosensor response decreasing due to the deprotonation of the GFP cell chromophore, and the amino acid chain of the chromophore becoming substantially disordered [34]. Hence, the optimum operational pH of the toxicity biosensor was determined to be pH 7.5.

### 3.4. Reproducibility and Long Term Stability Studies

**Figure 4:**
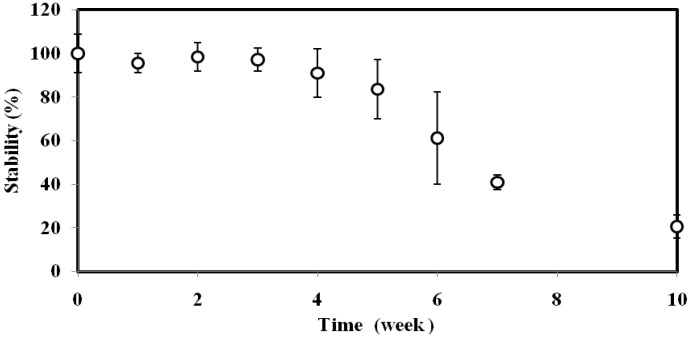
The stability profile of GFP *E. coli* biosensor at 0.8 Abs OD (600 nm) cell concentration, 4% alginate and 0.15 M CaCl_2_.

The reproducibility of the biosensor was found to be 3.6%–5.1% RSD (n = 8). The reproducibility of the biosensor was satisfactory, as the immobilized GFP *E. coli* cells were protected by a layer of gelatine alginate membrane to prevent the bacteria from extreme pH and temperature changes; the long term stability profile of the immobilized GFP *E. coli* over ten weeks is demonstrated in Figure 4. The biosensor response maintained its 100% stability for the first three weeks, and the biosensor response gradually declined thereafter. By week-10, the immobilized GFP *E. coli* loses 80% of its initial response due to the death of the immobilized bacteria from insufficient nutrient.

### 3.5. Biosensor Response towards Various Concentrations of Single Toxicants

Table 1 shows the toxicity biosensor and Microtox performance for the detection of various toxicants. The sensitivities of both GFP *E coli* biosensor and Microtox assay towards the various individual toxicants are in the order of Cu(II) > Cd(II) > Pb(II) > Zn(II) > Cr(IV) > Co(II) > Ni(II) > Ag(I) > Fe(III), and Cu(II) > Cd(II) = Pb(II) = Zn(II) > Cr(IV) > Co(II) > Ni(II) > Ag(I) > Fe(III), respectively. Generally, the proposed toxicity biosensor demonstrated various improvements compared to Microtox toxicity testing in terms of detection limit (LOD) and EC_50%_ in determining the heavy metals in water. The limit of detection was calculated from average blank responses (no toxicant) plus three standard deviation of the blank signal [35]. The biosensor showed highest sensitivity towards Cu(II) toxicity compared to other metal ions, because the thiol functional group of the cysteine amino acid possesses a high affinity to bind with Cu(II) ion to form Cu(II)-S complex and hydrogen (H^+^) ion [36]. According to Arias-Barreiro *et al.* [13], a study based on free GFP *E. coli* bacteria, the lowest LOD was obtained for Cu(II) ion at 0.1 μg/L compared with Cd(II) (1.0 μg/L), Pb(II) (6300 μg/L) and Zn(II) (1.0 μg/L) toxicities at 1 min, 3 min, and 5 min response times, respectively [13]. Based on Table 2, The proposed GFP *E. coli* biosensor for the detection of Cu(II), Cd(II), Pb(II), Zn(II), Cr(VI), Co(II), Ni(II), Ag(I) and Fe(III) toxicities showed improved performance with regard to LOD, dynamic range, and response times when compared with other reported microorganism-based toxicity methods e.g., optical biosensor [19], amperometry biosensor [37], microplate luminometry assay [38] and potentiometry biosensor [39]. Most of these methods utilize bacterial cells at high concentrations with long response time [38].

Table 3 reveals the EC_50_ values obtained from the immobilized GFP *E. coli* and previously reported EC_50_ values for Cu(II), Cd(II), Pb(II), Zn(II), Cr(VI), Co(II), Ni(II), Ag(I) and Fe(III) toxicants. The EC_50_ values estimated for Cu(II), Cd(II), Pb(II), Zn(II), Cr(VI), Co(II), Ni(II), Ag(I) and Fe(III) toxicities using the immobilized GFP *E. coli* were lower when compared with the EC_50_ values obtained using *A. fischeri* microbial cell [19]. The lower EC_50_ values obtained from immobilized GFP *E. coli* imply a more efficient toxicity biosensor, as lower amounts of metal ions are required to inhibit the biosensor. Furthermore, the biosensor based on immobilized GFP *E. coli* demonstrated a rapid response for the evaluation of heavy metal toxicity at two min response time, and it is faster when compared with other reported heavy metal assays.

**Table 1:** The comparison between green fluorescent protein (GFP) *E. coli* biosensor and Mictotox method for the detection of various single toxicants. The bacterial cell concentration used had an optical density of 0.8 Abs OD at 600 nm. The Ca-alginate membrane was prepared from 4% alginate and 0.15 M CaCl_2_.

Toxicants	Dynamic Range (µg/L)	LOD (µg/L)	Slopes (%RFU/µg·L^−1^)	EC_50_ (µg/L)	R^2^
**GFP cell biosensor, 2 min (n = 3)**					
Cu(II)	(0.05–1)	0.04	−45.081	0.9	0.98
Cd(II)	(0.50–10)	0.32	−5.015	8.9	0.99
Pb(II)	(0.70–20)	0.46	−2.564	17.4	0.99
Zn(II)	(5–100)	2.80	−0.506	84.4	0.99
Cr(VI)	(0.10–5) × 10^3^	1.00 × 10^2^	−0.009	4.5 × 10^3^	0.98
Co(II)	(0.50–7) × 10^3^	2.50 × 10^2^	−0.006	6.8 × 10^3^	0.99
Ni(II)	(0.70–10) × 10^3^	4.00 × 10^2^	−0.005	9.0 × 10^3^	0.98
Ag(II)	(1.00–20) × 10^3^	7.20 × 10^2^	−0.002	2.0 × 10^4^	0.99
Fe(III)	(5.00–70) × 10^3^	2.60 × 10^3^	0.001	6.4 × 10^4^	0.99
**Microtox assay, 15 min (n = 3)**					
Cu(II)	(0.03–2) × 10^3^	10.12	38.836× 10^−3^ *	1.1 × 10^3^	0.98
Cd(II)	(0.5–80) × 10^3^	0.42 × 10^3^	0.953 × 10^−3^ *	5.6 × 10^4^	0.99
Pb(II)	(0.5–80) × 10^3^	0.45 × 10^3^	0.810 × 10^−3^ *	6.1 × 10^3^	0.99
Zn(II)	(0.5–100) × 10^3^	0.46 × 10^3^	0.703 × 10^−3^ *	6.2 × 10^4^	0.99
Cr(VI)	(1–150) × 10^3^	0.50 × 10^3^	0.446 × 10^−3^ *	9.0 × 10^4^	0.98
Co(II)	(10–150) × 10^3^	5.60 × 10^3^	0.500 × 10^−3^ *	9.8 × 10^4^	0.99
Ni(II)	(1–120) × 10^3^	0.65 × 10^3^	0.549 × 10^−3^ *	6.5 × 10^4^	0.98
Ag(II)	(1–120) × 10^3^	0.52 × 10^3^	0.601 × 10^−3^ *	6.3 × 10^4^	0.96
Fe(III)	(15–150) × 10^3^	10.25 × 10^3^	0.504 × 10^−3^ *	9.5 × 10^4^	0.97

Note: * = Slope (inhibition (%)/µg·L^−1^).

**Table 2:** Comparison between the developed microbial biosensor performance and other microorganism-based biosensors for the detection of Cu(II), Cd(II), Pb(II), Zn(II), Cr(VI), Co(II), Ni(II), Ag(I) and Fe(III) toxicities.

Analyte	Biological Component	Immobilization Matrix	Dynamic Range (μg/L)	LOD (μg/L)	Time (min)	References
Cu(II)	*GFP E. coli*	Alginate film	(0.05–1)	4.0 × 10^−2^	2	This work
	*H. crispa*	Agar gel	(0.01–672) × 10^3^	6.0	20	[37]
	*A. fischeri*	Alginate microspheres	(0.1–2.0) × 10^2^	6.4	6	[19]
Cd(II)	*GFP E. coli*	Alginate film	(0.5–10)	0.32	2	This work
	*S. cereviceae*	Agar gel	(4.6–45.8) × 10^3^	1.83 × 10^3^	8	[37]
	*A. fischeri*	Alginate microspheres	(0.2–5.0) × 10^3^	1.6 × 10^2^	6	[19]
Pb(II)	*GFP E. coli*	Alginate film	(0.7–20)	0.36	2	This work
	*A. fischeri*	Alginate microspheres	(0.5–7) × 10^2^	0.5 × 10^2^	6	[19]
Zn(II)	*GFP E. coli*	Alginate film	(5–100)	2.80	2	This work
	*A. fischeri*	Alginate microspheres	(0.5–7) × 10^2^	0.32 × 10^3^	6	[19]
Cr(VI)	*GFP E. coli*	Alginate film	(0.1–5) × 10^3^	1.0 × 10^2^	2	This work
	*A.ferrooxidans*	Cellulose membrane	(0.02–118) × 10^3^	18	1	[36]
	*A. fischeri*	Alginate microspheres	(0.1–2 ) × 10^4^	1.0 × 10^3^	6	[19]
Co(II)	*GFP E. coli*	Alginate film	(0.5–7) × 10^3^	2.5 × 10^2^	2	This work
	*A. fischeri*	Alginate microspheres	(0.5–5.0) × 10^4^	1.7 × 10^3^	6	[19]
Ni(II)	*GFP E. coli*	Alginate film	(0.7–10) × 10^3^	4.2 × 10^2^	2	This work
	*B. sphaericus*	Whatman membrane	(2–40)	0.02	2	[38]
	*A. fischeri*	Alginate microspheres	(0.5–7) × 10^4^	2.8 × 10^3^	6	[19]
Ag(I)	*GFP E. coli*	Alginate film	(0.1–2) ×10^4^	7.2 × 10^2^	2	This work
	*A. fischeri*	Alginate microspheres	(0.2–7) × 10^4^	1.8 × 10^3^	6	[19]
Fe(III)	*GFP E. coli*	Alginate film	(5.0–70) × 10^3^	2.60 × 10^3^	2	This work
	*A. fischeri*	Alginate microspheres	(0.5–7) × 10^4^	0.31 × 10^4^	6	[19]

**Table 3:** The comparison between the developed toxicity biosensor and previously reported work for the estimation of EC_50_ values for Cu(II), Cd(II), Pb(II), Zn(II), Cr(VI), Co(II), Ni(II), Ag(I) and Fe(III) toxicities.

Heavy Metals and Incubation Times	This Work	Futra *et al.* [19]
Times (min)	2	6
Cu(II) (μg/L)	0.9	1.7 × 10^2^
Cd(II) (μg/L)	8.9	6.3 × 10^3^
Pb(II) (μg/L)	17.4	0.7 × 10^3^
Zn(II) (µg/L	84.4	6.0 × 10^2^
Cr(VI) (μg/L)	4.5 × 10^3^	1.8 × 10^4^
Co(II) (μg/L)	6.8 × 10^3^	6.6 × 10^4^
Ni(II) (μg/L)	9.0 × 10^3^	6.6 × 10^4^
Ag(I) (μg/L)	2.0 × 10^4^	6.0 × 10^4^
Fe(III) (µg/L)	6.4 × 10^4^	7.0 × 10^4^

### 3.6. Biosensor Response towards Combined Metals

Generally, the biosensor inhibition response showed antagonistic response towards various toxicity mixtures (Table 4). This response was due to the competitive reaction between various heavy metal ions for active sites (thiol functional group) of the GFP bioreceptor [8,27]. The competition held between the elements reduced the toxicity impact towards GFP *E. coli* biosensor compared to a single toxicant.

**Table 4:** The additive index (AI) values determined by the GFP *E. coli* biosensor for toxicity mixture of Cu(II), Cd(II), Pb(II) and Zn(II) at various concentration ratios.

Toxicant Mixture	AI	Toxicity Rate
(1:1 w/w)		
Pb(II) + Zn(II)	−0.41	Antagonistic
Cu(II) + Zn(II)	−3.73	Antagonistic
Cu(II) + Pb(II)	−1.72	Antagonistic
Cd(II) +Zn(II)	−1.62	Antagonistic
Cd(II) + Pb(II)	−2.94	Antagonistic
Cd(II) + Cu(II)	−1.97	Antagonistic
(2:1 w/w)		
Pb(II) + Zn(II)	−0.27	Antagonistic
Cu(II) + Zn(II)	−1.47	Antagonistic
Cu(II) + Pb(II)	−1.79	Antagonistic
Cd(II) +Zn(II)	−0.72	Antagonistic
Cd(II) + Pb(II)	−2.07	Antagonistic
Cd(II) + Cu(II)	−1.87	Antagonistic
(1:2 w/w)		
Pb(II) + Zn(II)	−0.878	Antagonistic
Cu(II) + Zn(II)	−1.64	Antagonistic
Cu(II) + Pb(II)	−2.00	Antagonistic
Cd(II) +Zn(II)	−0.75	Antagonistic
Cd(II) + Pb(II)	−1.58	Antagonistic
Cd(II) + Cu(II)	−2.88	Antagonistic
(1:1:1 to 1:1:1:1 w/w)		
Cu(II) + Cd(II) + Pb(II)	−1.984	Antagonistic
Cu(II) + Cd(II) + Zn(II)	−1.990	Antagonistic
Cd(II) + Pb(II) + Zn(II)	−3.186	Antagonistic
Cu(II) + Cd(II) + Pb(II) + Zn(II)	−6.033	Antagonistic

## 4. Conclusions

In this study, a toxicity biosensor based on GFP *E. coli* for the detection of single and combined heavy metals was successfully developed. The biosensor demonstrated promising performance to evaluate heavy metal toxicity in terms of dynamic range, detection limit (LOD), reproducibility, and repeatability. The high stability toxicity biosensor based on GFP *E. coli* was found to be sensitive for the evaluation of heavy metal toxicity, and it could provide a rapid response in two minutes. The biosensor demonstrated higher sensitivity in metal toxicity evaluation when compared to conventional Microtox assay.
